# Current State of Pediatric Urology Training: Resident Competency Expectations vs. Perceived Ability

**DOI:** 10.7759/cureus.95949

**Published:** 2025-11-02

**Authors:** Kerry Adler, Kaitlyn Sbrollini, Katie Yang, Arthur Chen, Kate Kraft, Michael Ernst

**Affiliations:** 1 Urology, Stony Brook University Hospital, Stony Brook, USA; 2 Urology, New York Medical College, Valhalla, USA; 3 Urology, University of Michigan, Ann Arbor, USA

**Keywords:** competency-based education, pediatric urology, procedural proficiency, resident autonomy, surgical education, urology curriculum

## Abstract

Objective: This study aimed to evaluate the expectations of program directors (PDs) and pediatric urologists regarding chief resident autonomy in core pediatric urology procedures and to compare these expectations with pediatric urologists’ observed resident performance.

Methods: Two national surveys were distributed: one to pediatric urologists through the Society of Pediatric Urology (SPU) and one to U.S. urology PDs. Both surveys evaluated expectations for chief resident performance across pediatric urology cases using the validated Zwisch scale. Only SPU surveys assessed observed resident performance.

Results: Survey response rates were 32% (SPU) and 33% (PDs). Although 96% of PDs reported a dedicated pediatric rotation in their programs, 60% of pediatric urologists spent ≤6 months working with residents. PDs held higher expectations than pediatric urologists for chief resident autonomy across all types of pediatric cases. Autonomy was expected by most for simple cases (e.g., orchiopexy, hydrocele repair), but less so for complex cases (e.g., hypospadias repair, pyeloplasty). Notably, observed resident proficiency reported by pediatric urologists consistently fell short of both their own and PDs' expectations.

Conclusions: Despite structured pediatric exposure, a gap remains between expected and actual resident proficiency in pediatric urology. Discrepancies between program directors' and pediatric urologists' expectations, especially for complex procedures, may reflect sub-specialization trends and varying exposure levels. Expanding pediatric training, adjusting expectations, or realigning residency goals with realistic future practice demands may be necessary.

## Introduction

Pediatric urology is a core component of resident education, assessed on the in-service exam and parts I and II of the American Board of Urology exam. In a study analyzing 2010 to 2018 pediatric case logs from graduating residents [1], the mean reported case volumes outpaced the minimum, with all urology residents meeting pediatric case minimums in 2018. Despite these promising numbers, actual resident competency at graduation may not reflect this data.

Despite the Accreditation Council for Graduate Medical Education (ACGME) to standardize and improve training throughout surgical residencies [1], preparation of residents for clinical practice or fellowship training remains subpar [2,3]. Urology program directors (PDs) have cited insufficient staffing, funding, and validated evaluation instruments as barriers to implementing ACGME core competencies [4]. Additionally, resident autonomy has declined as sub-specialization has deepened within the field at large. A 2019 study by Okhunov et al. investigated potential deficiencies in urology residency training programs in the United States, revealing discrepancies in perceptions of subspecialty exposure between PDs and residents [5]. Though pediatric-specific surgical skills were not assessed, competency with other surgical skills was rated differently by response groups, with many residents expressing a perceived lack of confidence in many complex procedures commonly encountered in the current practice of general urology. It is imperative to assess graduating residents' competencies in all aspects of urology specialty training. Innovation, such as the Competency-Based Medical Education's pilot novel evaluation application, is essential. The Society for Improvement in Medical Proficiency (SIMPL) application was piloted in one urology program and subsequently adopted by 17 more programs [6,7]. The standard of surgical competence must be clearly defined and consistently measured to ensure all trainees achieve readiness for independent practice, highlighting the need to expand efforts to understand specificities of training proficiencies across diverse procedures and institutions.

To this end, we wished to gain insight from PDs and pediatric urologists into the current state of pediatric urology curricula in US residencies. In the United States, urology residency is a five- to six-year program and encompasses mostly adult urology. However, urology residents typically rotate in pediatric urology during their mid-training years under the supervision of fellowship-trained pediatric urologists. The duration and intensity of pediatric exposure vary across institutions, and program directors may be either pediatric or general urologists, contributing to heterogeneity in resident experiences and expectations for procedural autonomy. We sought to identify gaps between expected and observed proficiency of chief residents from the perspective of PDs and pediatric urologists in multiple pediatric cases. The findings of two surveys offer a unique perspective on the current state of pediatric urology education and potential areas for improvement in clinical training.

## Materials and methods

Study design 

This was a cross-sectional survey study designed to evaluate expectations regarding graduating urology residents’ proficiency in pediatric urology procedures. Two parallel surveys were created: one for members of the Society of Pediatric Urology (SPU; Survey A, Appendix A) and one for urology residency PDs in the United States (Survey B, Appendix A). 

Sample size calculation 

We estimated an effect size of 25% (e.g., 45% vs. 20%) for complex surgical pediatric urology surgeries, such as proximal hypospadias repairs and pyeloplasty, and 10% (e.g., 90% vs. 80%) for more routine pediatric urology surgeries in autonomy expectations between program directors and pediatric urologists. This corresponded to required group sizes of approximately 59 and 216 participants, respectively, to achieve 80% power at α = 0.05. Sample size calculations were performed using the Statulator Sample Size Calculator for Comparing Two Independent Proportions (ss2P) tool (https://statulator.com). 

Sample characteristics 

Survey A was distributed to all active members of the SPU. Respondents were asked to provide demographic information, including gender, years in practice, American Urological Association (AUA) section, practice setting at a standalone children’s hospital, and employment type (academic vs. private practice). 

Survey B was distributed to all urology residency PDs in the United States. Demographic data collected included gender, AUA section, subspecialization, and years in the PD role. Program-level characteristics included the presence and post-graduate year (PGY) level of dedicated pediatric urology rotations, total time on pediatric urology, availability of elective opportunities beyond required blocks, criteria for resident assignment to pediatric cases, presence of a standalone children’s hospital, whether pediatric urologists were considered core faculty, and the year of residency in which most trainees complete the minimum case requirement in pediatric urology. 

Questionnaire 

The study team, composed of pediatric urologists and faculty involved in residency education, developed both questionnaires. Content was informed by prior literature on resident autonomy, case log requirements, and expert consensus within the group. The surveys were reviewed initially by two pediatric urology faculty for clarity and relevance, establishing face and content validity. The surveys were then approved by full authorship prior to distribution. 

Survey A consisted of approximately 16 items, including demographic questions and case-specific questions using the Zwisch scale to evaluate expectations for resident independence across common pediatric urology procedures. Survey B included approximately 17 items, covering demographic and program characteristics in addition to Zwisch scale-based assessments of resident proficiency in the same procedures. 

The Zwisch scale was incorporated into both surveys to guide respondents’ judgments of chief resident performance and to standardize assessment of resident abilities. Originally developed to support graded autonomy, the Zwisch scale helps faculty gauge trainee progress toward surgical independence [8]. It measures the level of guidance required during critical portions of a procedure on a four-point scale: 1 (“Show and Tell”), 2 (“Active Help”), 3 (“Passive Help”), and 4 (“Supervision Only”). The Zwisch scale has been validated as a method of assessing resident operative autonomy [9] and has also been used in large national studies evaluating resident autonomy and readiness for independent practice in general surgery [10].

Although the full surveys were not formally validated, they incorporated a previously validated framework (Zwisch) and underwent expert review before distribution. 

The primary outcomes were respondents' expectations regarding the level of independence graduating residents should achieve in performing selected pediatric urology procedures. Cases included distal hypospadias, proximal hypospadias, circumcision revision, inguinal hydrocele repair, inguinal orchiopexy, testicular torsion orchiopexy, ureteral reimplant, and pyeloplasty. Respondents indicated whether residents should be capable of performing each procedure independently, with passive help, or under active supervision. 

Survey administration 

Both surveys were administered electronically using REDCap (Research Electronic Data Capture), a secure web-based platform for research data collection developed at Vanderbilt University, Nashville, TN. An initial invitation email was sent, followed by two reminder emails spaced approximately two weeks apart to improve response rates. The surveys were available for a total of six weeks. Participation was voluntary, and informed consent was implied by survey completion. No identifying information was collected. Average completion time was estimated at 10-15 minutes. 

Data collection and analysis 

All survey responses were collected directly in REDCap and exported for analysis. Responses were automatically de-identified. Incomplete responses were excluded. 

Descriptive statistics were generated for demographic and program characteristics. Responses from SPU members (Survey A) and PDs (Survey B) were compared using chi-square analysis for categorical variables. Statistical significance was defined as p < 0.05. Data were analyzed using IBM SPSS Statistics, version 28.0 (IBM Corp., Armonk, NY). 

## Results

**Figure 1:**
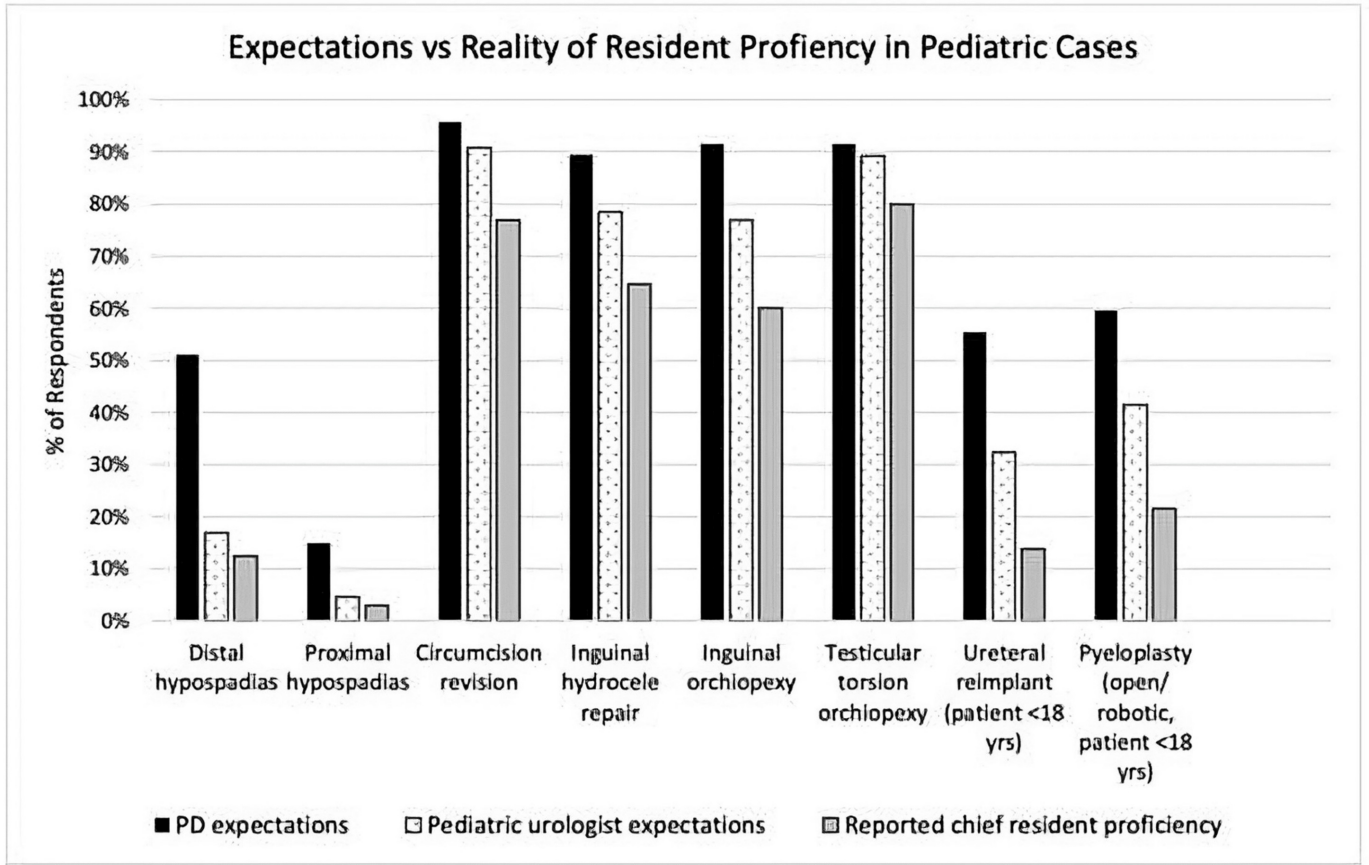
Alignment of the expectations of pediatric urologists and PDs with actual performance of chief residents in pediatric cases PDs: program directors

Survey A was sent to all SPU members; of the 201 recipients who opened the email, 65 responded (32% response rate). One reminder email was sent. Most participants (61.5%) worked with residents for ≤6 months during the residents’ training. Respondents worked with residents during PGY-3 (55.4%) most commonly and least during PGY-5 (6.2%) (Table 1). Most participants did not indicate working with fellows (68%). Pediatric urologists expected chief residents to achieve autonomy (at the level of passive help or supervision only) in most cases included in Survey A, including circumcision revision (90.8%), inguinal hydrocele repair (78.5%), inguinal orchiopexy (76.5%), and testicular torsion orchiopexy (89.2%) (Figure 1). It was largely not expected that chief residents would be operating autonomously on a case of distal (16.9%) or proximal hypospadias (4.6%), ureteral reimplant (32.3%), or pyeloplasty (41.5%). Observed autonomy fell below expectations of both PDs and pediatric urologists for all procedures (Figure 1).

There were no significant associations between working with fellows, years in practice, AUA section, or time spent with residents, and expectations for chief residents (p>0.05). There was a strong direct correlation between expectations of pediatric urologists and observed performance of chief residents (r=0.98, p<0.05). The primary outcome, chief resident autonomy in specific pediatric cases, fell below the expectations of pediatric urologists working with residents.

Survey B, sent to 143 urology PDs, had a 33% response rate (47). Nearly all programs have a dedicated pediatric urology rotation (95.7%) (Table 1), with most being less than six months (42.5%) and performed at a standalone children's hospital. Most rotations were carried out during PGY-2 (30.9%), PGY-3 (27.2%), and PGY-4 (23.4%) years. Additional non-OR learning opportunities were widely available to residents, including pediatric urology clinic, pediatric urodynamics, pediatric journal clubs, and didactic conferences. Less than half of PDs reported office-based circumcision as an opportunity available to residents (44.7%).

**Table 1:** Insights from PDs into pediatric urology curriculum at US urology residencies PDs: program directors; PGY: post-graduate year; Uro: urology

Insights from PDs into pediatric urology curriculum at US urology residencies		
Years spent as a PD	n	%
1-5 years	21	44.7%
6-10 years	13	27.7%
>10 years	12	25.5%
No response	1	2.1%
Inclusion of a dedicated rotation in pediatric urology?	n	%
Yes	45	95.7%
No	1	2.1%
No response	1	2.1%
Total months spent in dedicated pediatric rotations	n	%
<6 months	20	42.5%
6 months	14	29.8%
>6 months	11	23.4%
No response	2	4.3%
PGY clinical year(s) during which the dedicated rotation(s) were performed	n	%
Uro-1	6	7.4%
Uro-2	25	30.9%
Uro-3	22	27.2%
Uro-4	27	33.3%
Uro-5	1	1.2%
Resident learning opportunities	n	%
Pediatric didactic conferences	40	85.1%
Pediatric urology clinic	39	83%
Pediatric urodynamics	37	78.7%
Pediatric journal clubs	32	68.1%
Office-based circumcisions	21	44.7%

Autonomous performance was expected in distal hypospadias (51.1%), circumcision revision (95.7%), inguinal orchiopexy (91.5%), testicular torsion orchiopexy (91.5%), pyeloplasty (59.6%), and ureteral reimplant (55.3%). Neither Survey A nor Survey B distinguished between pyeloplasty performed open versus robotically. We did not find a significant relationship between years spent as a PD and expectations for chief resident proficiency (p=0.356). The actual proficiency of chief residents was not assessed by Survey B (Table 2).

**Table 2:** Insights from pediatric urologists into pediatric curriculum at US urology residencies PGY: post-graduate year; Uro: urology

Insights from pediatric urologists into pediatric curriculum at US urology residencies		
Primary employment type	n	%
Academic	44	67.7%
Hospital-employed	13	20.0%
Self-employed	6	9.2%
Other employment type	1	1.5%
No response	1	1.5%
Years practicing as an attending physician	n	%
1-10	14	21.5%
11-20	17	26.1%
21+	33	50.7%
No response	1	1.5%
Months spent working with residents during their rotations	n	%
≤6	40	61.5%
7+	19	29.2%
No response	6	9.2%
Do you work with urology fellows?	n	%
Yes	21	32%
No	44	68%
During which PGY clinical year(s) do residents generally work with you?	n	%
Uro-1	16	24.6%
Uro-2	30	46.2%
Uro-3	36	55.4%
Uro-4	28	43.1%
Uro-5	4	6.2%

For each pediatric surgical case listed by Surveys A and B, PD expectations of chief residents were higher than expectations held by pediatric urologists (Table 3, Figure 1).

**Table 3:** Expectations of PDs and pediatric urologists vs. reported chief resident proficiency PDs: program directors

Expectations of PDs and pediatric urologists vs. reported chief resident proficiency			
Pediatric urology cases	PD expectations n (%)	Pediatric urologist expectations n (%)	Reported chief resident proficiency n (%)
Distal hypospadias	24 (51.1%)	11 (16.9%)	8 (12.4%)
Proximal hypospadias	7 (14.9%)	2 (4.6%)	2 (3%)
Circumcision revision	45 (95.7%)	59 (90.8%)	50 (76.9%)
Inguinal hydrocele repair	42 (89.4%)	51 (78.5%)	42 (64.6%)
Inguinal orchiopexy	43 (91.5%)	50 (76.9%)	39 (60%)
Testicular torsion orchiopexy	43 (91.5%)	58 (89.2%)	52 (80%)
Ureteral reimplant (patient <18 yrs)	26 (55.3%)	21 (32.3%)	9 (13.8%)
Pyeloplasty (open/robotic, patient <18 yrs)	28 (59.6%)	27 (41.5%)	12 (21.5%)
PD n=47, pediatric urologist n=65; chief resident proficiency is based on pediatric urologist observation of resident performance			

## Discussion

Our results suggest that the pediatric urology curriculum is robust and dynamic across most US urology residency programs with dedicated rotations and access to didactics, clinics, and journal clubs. However, these surveys also suggest that there is a gap between expected and actual resident proficiency. Both PDs and pediatric urologists anticipated greater chief resident autonomy in key pediatric cases than what was observed.

Interestingly, we found that PDs held higher expectations across the board for chief resident performance than did pediatric urologists. This held true for both “simple” and more “complex” cases. This may reflect the fact that only 7/47 (15%) of surveyed PDs were self-declared “pediatric urologists.” PDs in non-pediatric fields may underestimate the complexity of select pediatric cases compared to pediatric urologists [11]. Current PDs may have trained in environments where pediatric cases were practiced more widely by those outside the subspecialty. As urology becomes more subspecialized, it may be more difficult for today’s residents to achieve the same “all-around” competency as in the past [12]. Indeed, PD expectations are more closely aligned with those of pediatric urologists for more common pediatric cases, such as testicular torsion orchiopexy, inguinal orchiopexy, inguinal hydrocele repair, and circumcision revision, procedures with adult counterparts (adult torsions, adult circumcisions, and adult hydrocelectomies) commonly practiced by generalists [13]. If PDs expect resident autonomy in more complex pediatric cases, additional pediatric training may be needed, though this would require balancing time away from other subspecialties and weighing the benefit to future practice. 

For complex cases like hypospadias repair, pyeloplasty, and ureteral reimplantation, pediatric urologists held lower expectations overall of chief residents compared to PDs. This likely reflects their own fellowship training, during which they gain comfort with such procedures [14]. Therefore, although they hold lower expectations for chief residents, this may not entirely reflect resident performance and may, in fact, just be a realistic expectation based on personal experience. Additionally, these surgeries often involve more direct attending involvement, limiting resident autonomy and thereby influencing Zwisch scale ratings of resident autonomy. Masterson et al. (2023) also addressed expectations for autonomy, investigating attitudes toward resident autonomy in various types of hypospadias repairs using the Zwisch scale [15]. Interestingly, almost all (98%) of respondents from the SPU felt trainees should not be performing hypospadias repair independently in practice without completing a pediatric fellowship. Studies like this, and our own, indicate that pediatric urologists likely hold strict expectations of their chief residents and are less likely to judge them capable of autonomy for more complex procedures. 

For all cases, chief residents fell short of expectations held by pediatric urologists. Interestingly, most pediatric urologists primarily worked with junior residents, with only 4% reporting regular involvement with chiefs. This may afford fewer opportunities to train junior residents to proficiency that matches expectations for more senior residents. Moreover, 60% of pediatric urologists reported working less than or equal to six months with any level of resident; this accounted for either a pediatrics rotation or the total time spent with residents throughout residency training. Since many pediatric urologists felt that chief residents did not meet their expectations, it is possible that six months is not enough time to adequately train residents to autonomy in select pediatric cases. To rectify this, programs may expand the time residents spend on pediatric rotations or ensure that the months spent doing pediatric cases are allotted to more senior PGY years so that chiefs may reach the competency expected of them by both PDs and pediatric urology staff. 

Another reason for the discrepancy may be a mismatch between the goals that pediatric urologists set for chief residents and the opportunities that residents are afforded. Importantly, our study did not assess pediatric case volume, which likely varies across programs and may influence both exposure and expectations [13]. Pediatric clinical training may be unique due to the dedicated rotational structure of the clinical specialty rather than integration into training more broadly. Mickelson et al. (2008) examined the competencies of Canadian urology residents upon graduation in pediatric cases from the perspective of senior urology residents, residency PDs, and Pediatric Urologists of Canada (PUC) [11]. This study assessed the perceived competency of Canadian urology residents in 23 pediatric procedures across varying complexity. Residents averaged 5.4 months of pediatric exposure over five years. This study found that PDs and residents rated trainee competency significantly higher than members of the PUC in the two less complex categories, but in the most complex category, there was no statistically significant difference between participant groups. In the least complex category, trainee competency was confirmed by 85% of PDs, 75% of senior residents, and 67% of PUCs (p<0.05). In the middle category, competency was confirmed by 60% of PDs, 50% of senior residents, and 37% of PUCs (p<0.05). This study’s investigation revealed curious results that warrant similar scrutiny of training programs in the United States. 

One limitation of our study is sample size, which, while sufficient for significant results, certainly may not capture the entire country’s perception of chief resident competency in pediatric cases. Additionally, the subjective manner of our surveys opens the results up to personal and professional biases in all participants. The nature of this study, inquiring about ‘chief residents’ as a demographic group rather than as individual residents, is limited by potential recall bias. Participants may answer survey questions while retroactively assessing the capabilities of chief residents of years past. Other data points that could have been collected to illuminate nuances in the data include whether a resident intends to go on to a pediatric urology fellowship or further training in any fellowship program, as well as all participants’ projected likelihood of performing the cases assessed in their practice as an attending-level physician. The type of practice a resident is planning to enter, either private practice or academic, as well as the geographical nature of their role after graduating from residency, may also affect the results of the survey, as well as the importance of the results. 

As shown by Masterson et al., pediatric urologists often view more complex pediatric urology procedures, such as distal hypospadias, as inappropriate for autonomous resident performance [15]. We therefore anticipated larger differences in expectations for these procedures than for more routine operations. However, given the finite number of pediatric urologists and PDs available to survey, our study may have been underpowered to detect smaller differences in autonomy expectations for common procedures where baseline agreement is high. This introduces a risk of type II error and may limit our ability to draw conclusions about more routine pediatric cases. While limited in size, our sample captures a substantial proportion of the accessible population, providing valuable insight into autonomy expectations across pediatric urologists and PDs. 

Although we surveyed both PDs and pediatric urologists, the residents remain a crucial part of the equation. Future studies would benefit from gauging how chief residents view their training experience in pediatrics. Only a small proportion of urology residents in the United States pursue fellowship training in pediatric urology, approximately 4%-7% of graduating residents each year [16]. This limited sub-specialization may contribute to reduced resident exposure to pediatric cases during training. It is also possible that the amount of time or the level of PGYs dedicated to pediatrics is felt to be sufficient by residents themselves or reflects resident input into their program structure. Furthermore, resident expectations for their own performance in pediatric cases may not align with those of PDs or pediatric urology staff [5]. Instead of resident capabilities on pediatric cases improving, perhaps expectations need to shift. If residents are not expected to perform these cases independently in practice, then are we seeing a true performance gap, or should residency training shift the focus to a reasonable expectation of future practice? It is critical to understand the resident perspective, as well as the changing climate of urology, to evaluate how to best incorporate pediatrics into the training of urology residents. 

## Conclusions

The gaps revealed by this study emphasize the need for more targeted training, feedback, and support to bridge the divide between what is expected and where residents are truly performing. Though the perceptions of our study participants are revealing, further investigation should consider potential objective measures of resident competency and resident self-assessment.

Understanding and investigating the autonomous competencies of chief residents should be rigorously assessed as the field of urology continues to grow and evolve. Other subspecialties of urology may consider pursuing endeavors like our study to navigate their own unique challenges during residency training.

## Appendices

Appendix A

Survey A: Pediatric Urology Curriculum: Survey of Pediatric Urologists

What gender do you identify as?

○ Female ○ Male ○ Nonbinary ○ Prefer not to answer

How many years have you been practicing as an attending physician?

○ 1-5 years ○ 6-10 years ○ 11-15 years ○ 16-20 years ○ 21-25 years ○ 26+ years

Which AUA section do you belong to?

○ Northeastern Section ○ New England Section ○ New York Section ○ Mid-Atlantic Section ○ North Central Section ○ Southeastern Section ○ South Central Section ○ Western Section

Do you work at a standalone children's hospital (operating rooms that are geographically separate from the location of other urology residency rotations)?

○ Yes ○ No 

What is your primary employment type?

○ Self-employed ○ Academic ○ Hospital-employed ○ Other employment type

Do you work with urology residents?

○ Yes ○ No

Do you work with urology fellows?

○ Yes ○ No

How many years have you been working with residents?

○ 0-5 years ○ 6-10 years ○ 11-15 years ○ 16+ years

How many months do you work with a resident during their residency? (either an exact number if it is a  rotation, or an estimated number if no specific rotation)

○ 1-2 months ○ 3-4 months ○ 5-6 months ○ 7-8 months ○ 8-9 months ○ 9-10 months ○ 11-12 months ○ 13+ months

Do the residents you work with have a dedicated pediatrics rotation (or rotations)? 

○ Yes ○ No ○ Unsure

If the residents you work with have a dedicated pediatrics rotation (or rotations), can the residents choose to do additional pediatric cases outside of the rotation(s)?

○ Yes ○ No ○ Unsure

During which PGY clinical year(s) do residents generally work with you? Check all that apply 

□ Uro-1 □ Uro-2 □ Uro-3 □ Uro-4 □ Uro-5

Who decides which pediatric urology cases an individual resident is assigned to? (select all that apply)

□ Program Chair □ Program Director □ Fellow, chief resident, or other resident □ The individual resident □ Pediatric urology faculty □ Unsure/unknown

What criteria are used to determine resident assignments to pediatric urology cases? (select all that apply) 

□ Complexity of case □ Ability of individual resident □ PGY year □ Availability of resident coverage □ Unsure/unknown Which of the following experiences do the residents you work with have the opportunity to participate in?  (select all that apply) 

□ Pediatric urology clinic □ Pediatric urodynamics □ Pediatric journal clubs □ Pediatric didactic conferences □ Office-based circumcisions □ All of the above □ None of the above

The Zwisch scale was designed to guide faculty to develop graduated autonomy for their learners during training. It acts as an assessment tool, allowing faculty to understand where each trainee stands in their progression towards independence, and provides teaching strategies to help them progress. For the pediatric urology cases below, please indicate which cases you EXPECT the average chief resident at the end of residency to be able to perform at the level of passive help or supervision only (select all that apply).

□ Distal hypospadias □ Proximal hypospadias □ Circumcision revision □ Inguinal hydrocele repair □ Inguinal orchiopexy □ Testicular torsion orchiopexy □ Ureteral reimplant (patient < 18 years old) □ Pyeloplasty (open or robotic, patient < 18 years old) 

The Zwisch scale was designed to guide faculty to develop graduated autonomy for their learners during training. It acts as an assessment tool, allowing faculty to understand where each trainee stands in their progression towards independence, and provides teaching strategies to help them progress. For the pediatric urology cases below, please indicate which cases the average residency graduate you worked with over the past 5 years (at your program/institution/hospital) CAN perform at the end of residency at the level of passive help or supervision only (select all that apply).

□ Distal hypospadias □ Proximal hypospadias □ Circumcision revision □ Inguinal hydrocele repair □ Inguinal orchiopexy □ Testicular torsion orchiopexy □ Ureteral reimplant (patient < 18 years old) □ Pyeloplasty (open or robotic, patient < 18 years old) 

Appendix B

Survey B: Pediatric Urology Curriculum: Survey of Program Directors

What gender do you identify as?

○ Female ○ Male ○ Nonbinary ○ Prefer not to answer

Which AUA section do you belong to?

○ Northeastern Section ○ New England Section ○ New York Section ○ Mid-Atlantic Section ○ North Central Section ○ Southeastern Section ○ South Central Section ○ Western Section

What is your primary area of specialization?

○ General Urology ○ Andrology/Infertility ○ Endourology/Minimally Invasive Urology ○ Female Urology ○ Pediatric Urology ○ Reconstructive Urology ○ Urologic Oncology ○ Other

How many years have you been practicing as a Program Director?

○ 1-5 years ○ 6-10 years ○ 11-15 years ○ 16-20 years ○ 21-25 years ○ 26-30 years ○ >31 years

Does your residency program include a dedicated rotation in pediatric urology?

○ Yes ○ No

How many total months (weeks) are spent in these 1-month dedicated pediatric rotation(s)?

○ 1 month (4 weeks) ○ 2 months (8 weeks) ○ 3 months (12 weeks) ○ 4 months (16 weeks) ○ 5 months (20 weeks) ○ 6 months (24 weeks) ○ > 6 months (>24 weeks)

During which PGY clinical year(s) is/are the dedicated staff generally work with you? Check all that apply 

□ Uro-1 □ Uro-2 □ Uro-3 □ Uro-4 □ Uro-5

Are the dedicated pediatric rotations performed at a standalone children's hospital (operating rooms that are geographically separated from the location of other urology residency rotations)?

○ Yes ○ No

Is there an opportunity for residents to do an additional pediatric urology rotation or pediatric urology cases outside of their dedicated rotation blocks?

○ Yes ○ No

During which PGY clinical year(s) is/are residents performing pediatric urology cases? (select all that apply)

□ Uro-1 □ Uro-2 □ Uro-3 □ Uro-4 □ Uro-5

Who decides which pediatric urology cases an individual resident is assigned to? (select all that apply)

□ Program Chair □ Program Director □ Fellow, chief resident, or other resident □ The individual resident □ Pediatric urology faculty □ Unsure/unknown

What criteria are used to determine resident assignments to pediatric urology cases? (select all that apply)

□ Complexity of case □ Ability of individual resident □ PGY year □ Availability of resident coverage □ Unsure/unknown

Are pediatric urology cases performed at a standalone children's hospital (operating rooms that are geographically separated from the location of other urology residency rotations)?

○ Yes ○ No

During which PGY clinical year(s) is/are residents performing pediatric urology cases? (select all that apply)

○ Uro-1 ○ Uro-2 ○ Uro-3 ○ Uro-4 ○ Uro-5

Are the pediatric urologists who work with residents also core faculty within your department?

○ Yes ○ No

Which of the following experiences do your residents have the opportunity to participate in? (select all that apply)

□ Pediatric urology clinic □ Pediatric urodynamics □ Pediatric journal clubs □ Pediatric didactic conferences □ Office-based circumcisions □ All of the above □ None of the above

The Zwisch scale was designed to guide faculty to develop graduated autonomy for their learners during training. It acts as an assessment tool, allowing faculty to understand where each trainee stands in their progression towards independence, and provides teaching strategies to help them progress. For the pediatric urology cases below, please indicate which cases you EXPECT the average chief resident at the end of residency to be able to perform at the level of passive help or supervision only (select all that apply).

□ Distal hypospadias □ Proximal hypospadias □ Circumcision revision □ Inguinal hydrocele repair □ Inguinal orchiopexy □ Testicular torsion orchiopexy □ Ureteral reimplant (patient < 18 years old) □ Pyeloplasty (open or robotic, patient < 18 years old)
